# The duty to care and nurses’ well-being during a pandemic

**DOI:** 10.1177/09697330211041746

**Published:** 2022-02-10

**Authors:** C Amparo Muñoz-Rubilar, Carolina Pezoa Carrillos, Ingunn Pernille Mundal, Carlos De las Cuevas, Mariela Loreto Lara-Cabrera

**Affiliations:** Universidad Central de Chile, Chile; Molde University College, Norway; Møre og Romsdal Health Trust, Norway; Department of Internal Medicine, Dermatology and Psychiatry, University of La Laguna, San Cristóbal de La Laguna, Spain and Instituto Universitario de Neurociencias (IUNE), Universidad de La Laguna, Canary Islands, Spain.; St Olav’s Hospital University, Norway; Norwegian University of Science and Technology (NTNU), Norway

**Keywords:** Attitudes of health personnel, COVID-19 pandemic, duty to care, ethical issues nurses, psychological well-being, willingness to work

## Abstract

**Background:**

The coronavirus disease 2019 pandemic is impacting the delivery of healthcare worldwide, creating dilemmas related to the duty to care. Although understanding the ethical dilemmas about the duty to care among nurses is necessary to allow effective preparation, few studies have explored these concerns.

**Aim:**

This study aimed to identify the ethical dilemmas among clinical nurses in Spain and Chile. It primarily aimed to (1) identify nurses’ agreement with the duty to care despite high risks for themselves and/or their families, (2) describe nurses’ well-being and (3) describe the associations between well-being and the duty to care.

**Research design:**

Cross-sectional self-reported anonymous data were collected between May and June 2020 via electronic survey distribution (snowball sampling).

**Ethical considerations:**

The Institutional Ethical Review Committees in both countries approved the study (CHUC_2020_33 and 27/2020).

**Findings:**

In total, 345 clinical nurses answered the primary question about the duty to care for the sick. Although in the total sample 77.4% agreed they have a duty to care for the sick, significant differences were found between the Spanish and Chilean samples. Overall, 53.6% of the nurses reported low levels of well-being; however, among those reporting low well-being, statistically significant differences were found between Spanish and Chilean nurses as 19.4% and 37.8%, respectively, disagreed with the statement regarding the duty to care.

**Discussion:**

Participants in both countries reported several ethical dilemmas, safety fears, consequent stress and low well-being. These results suggest that prompt actions are required to address nurses’ ethical concerns, as they might affect their willingness to work and psychological well-being.

**Conclusion:**

Our findings shed light on the ethical dilemmas nurses are facing related to the duty to care. Not only has the coronavirus disease 2019 pandemic given rise to ethical challenges, but it has also affected nurses’ well-being and willingness to work during a pandemic.

## Introduction

Healthcare workers (HCWs) at the forefront of providing aid during pandemic outbreaks are exposed to risk, facing demanding professional responsibilities^1^ and uncertainties concerning their principal duty to care.^2^ Especially in times of crisis, and when there is a perceived high risk of infection, ethical concerns are visible in the case of public healthcare sectors. Previous studies have reported the following concerns among healthcare professionals during pandemics: fear of infection,^3^ perceived risk,^4,5^ concerns regarding work conditions,^6^ perceived stigma,^7,8^ low emotional well-being^9^ and psychosocial distress.^3,6,10^ The global public healthcare system has faced a scarcity of resources, and concerns regarding the risk of transmitting the severe acute respiratory syndrome coronavirus 2 (SARS-CoV-2) virus, due to a lack of personal protective equipment (PPE).^11,12^ For instance, Spain and Chile are two countries that have been severely affected by the coronavirus disease 2019 (COVID-19) pandemic, in Europe and South America, respectively. The virus was first confirmed to have spread to Spain on 31 January 2020, when a German tourist tested positive for SARS-CoV-2 in La Gomera, in the Canary Islands. Eventually, a nationwide lockdown was imposed on 14 March 2020. In May 2020, Spain became Europe’s next epicentre of the contagion, being severely affected by COVID-19; specifically, the pandemic caused chaos in the healthcare system, as more than 20% of those who contracted the virus were healthcare professionals.^13^ In June 2021, there have been a total of 3,729,458 Spanish cases, including 80,465 reported deaths as per World Health Organization (WHO).^14^ In Chile, since the detection of the first case on 3 March 2020, there have been a total of 1,468,992 cases. On 4 June 2021, the WHO reported that there have been a total of 30,579 deaths in Chile.^14^ In addition to several challenges due to the pandemic,^13,15^ both countries have reported socio-economic challenges that have a priori impacted health services.^13,16^ During the current pandemic, nurses have had to struggle with limited access to essential PPE and fears for their own safety. Nonetheless, while rarely documented, fears for one’s own safety, and concerns regarding the duty to care, can create ethical dilemmas for nurses.^4,5^ However, studies identifying ethical challenges among nurses working in countries with diverging welfare states, such as Spain and Chile, are limited.

## Background

Professional codes of ethics for clinical practice consist of values, duties, rights and responsibilities regulated by national legislation and international agreements and detailed in professional codes.^17^ The duty to care is among the most fundamental ethical obligation of healthcare providers. Although the duty is rooted in the fiduciary nature of the health professions in which the interests of the patient should take priority over other considerations, including a risk to their own health and life,^18^ nurses must balance their perceived duty to care against their perceived risk of harm.^19^ Ethical concerns related to the duty to care for patients can also conflict with the duty to care for one’s own family.^20^ A study conducted in England simulating an influenza pandemic scenario revealed that doctors and nurses agreed they have a duty to care for patients even when they are putting themselves into high-risk situations. However, respondents’ sense of duty to work might be conflicted with their sense of duty to their families.^21^ Findings from similar studies indicated that HCWs’ attitudes and beliefs about pandemics influence their willingness to work.^22,23^

During the current global public health crisis caused by the rapid spread of the SARS-CoV-2 virus, priority-setting dilemmas^24^ and ethical concerns due to the scarcity of resources^20,25^ have been reported among nurses and HCWs. A recent study aiming to explore the ethical dilemmas of Palestinian HCWs during the early COVID-19 pandemic^26^ found that almost 25% of surveyed HCWs were not willing to work during the pandemic. However, qualitative work from the same Palestinian study also reported that unwillingness did not preclude study participants from working.^26^ An online survey among physicians in Bangladesh^27^ reported that 8.9% of the participants were not willing to work during the pandemic, and another 21.4% indicated they were unsure of their willingness. Concern for family and risk of transmitting the infection to family members have been reported as major barriers of working during the pandemic,^27,28^ affecting the psychological well-being of nurses.^28^

Evidence suggests that nurses and HCWs may be at higher risk of adverse mental health outcomes during this pandemic,^29^ as caring for patients during an epidemic/pandemic negatively affects psychological well-being.^28,30^ As difficult work conditions might negatively impact the ethical concerns regarding the duty to care,^11,12^ identifying these rising concerns among nurses is important. A cross-sectional study^31^ examined how Israeli nurses responded to ethical dilemmas and tension during the COVID-19 outbreak and found that nurses reported conflicting values. Specifically, they found that most nurses (74.7%) do not believe they have the right to refuse to treat certain patients during the COVID-19 outbreak, and more than half (63.7%) agreed that neither a registered nurse nor an intern has the right to refuse to treat patients who may place them at risk.^31^ Another descriptive study among registered nurses working in hospitals in Israel found that when faced with a threat to personal safety or security, nurses might not be willing to work as usual.^32^ However, there is a paucity of empirical research in examining nurses’ agreement with the duty to care in the face of possible high risks for themselves or their families. Furthermore, no international comparative studies have yet been published in which ethical dilemmas related to the duty to care for the sick have been identified. Therefore, this study aimed to investigate the ethical dilemmas faced by nurses while working during the COVID-19 outbreak in Spain and Chile.

## Study aims

This study aimed to (1) identify nurses’ agreement with the duty to care despite high risks for themselves and/or their families, (2) describe nurses’ well-being and (3) describe the associations between well-being and duty to care. Secondary aims were to describe the nurses’ confidence, beliefs about managing COVID-19, fears and ethical concerns about a pandemic.

## Methods

### Design and recruitment

This research employed a cross-sectional descriptive design. The nursing schools’ management, nurse educators and the teaching staff at two universities sent email invitations to nurses working in hospitals. In addition, two associations of nurses freely posted the survey on their webpage to recruit nurses. The data were collected from 7 May to 28 June 2020. The email invitation included information about the study, anonymisation procedures and an electronic link to the online survey. Participants were also informed that their involvement was voluntary. Potential participants were asked to forward the web link to the online survey to other networks and via social media. The parameters were set to refuse multiple responses from the same Internet Protocol (IP) address to prevent duplication from the same participants.

In the survey, nurses were asked about their current working position, that is, whether they were involved with clinical, academic or administrative work, or were working with nursing education at the university. Nurses were eligible for this study if they self-reported that they (1) were employed in a hospital and (2) worked as staff clinical nurses during the COVID-19 pandemic.

### Survey measures

The questionnaire was composed of three parts. The first part assessed nurses’ confidence regarding managing COVID-19, fear and concerns of being infected, and how the COVID-19 pandemic affected the level of stress. The second part contained questionnaires to assess beliefs and attitudes towards a pandemic and self-reported psychological well-being. The third part contained participants’ basic demographic information.

#### Outcome variable

The primary ethical statement selected a priori was used to identify nurses’ agreement or disagreement with the duty to care for the sick. This was done with a single item: ‘Doctors and nurses have a duty to care for the sick, even when there are high risks for themselves or their families’.

The Spanish version of the questionnaire, Beliefs and Attitudes of Health Workers Toward a Pandemic, was used to assess dilemmas related to the duty to take care for the sick. The adapted Spanish version of the questionnaire^21,23^ included 15 questions to evaluate the informants’ ideas and experiences with pandemics. The instructions asked respondents to agree or disagree with different statements regarding ethics.

The well-being measurement tool used was the 5-item WHO-5 Well-Being Index. The validated Spanish version of the questionnaire included five items that explore the subjective well-being of the participants.^33^ Participants were asked to rate their agreement over the previous 2 weeks. Each item is rated on a 6-point scale from ‘all of the time’ to ‘at no time’. Total scores could range from 0 to 25, with higher scores representing higher perceived levels of well-being. A score below 13 indicates poor well-being and is an indication of requiring further evaluation.

### Statistical analysis

The descriptive analysis involved frequencies and percentages for categorical variables, and means and standard deviations for continuous variables. Chi-square and Fisher’s exact tests were used to compare ethical statements. The Fisher’s exact test was used when the data contained only a few observations in one of the cells (e.g. less than five). The primary outcome was measured with the statement ‘Doctors and nurses have a duty to care for the sick, even when there are high risks for themselves or their families’. This was calculated as the percentage reporting agreement and disagreement with the statement.

To evaluate the associations between well-being and agreement with the duty to care, the level of well-being was also coded as a dichotomous answer: low or high well-being. Differences within and between groups of nurses from the two countries were investigated, using chi-square tests, with a *p* < 0.05 being accepted as statistically significant. Statistical analyses were performed using SPSS v. 21.0 for Windows (IBM, Armonk, NY, USA).

### Ethical considerations

The study was reviewed and approved by the Institutional Ethical Review Committees in Spain (CHUC_2020_33) and Chile (27/2020). Following approval, the data were collected. The purpose of the study was explained to participants, and they had to sign an online informed consent prior to participation. It was not possible to participate in the survey without indicating that the information about the study has been read. By clicking ‘I agree’, the participants indicated they were at least 18 years old, were currently employed nurses, had read the consent form and agreed to participate in the research study. The statements of ethical standards in the Declaration of Helsinki were followed. While there were no direct harms by answering the survey, some of the participants could experience moral distress or stress. Therefore, we provided our names, email and contact information in the invitation letter. Confidentiality and anonymity were maintained by not asking for the participants’ names or other direct identifiers, such as address or other personal information, that could connect the data to the individuals who provided it. No identification list was created.

## Results

### Descriptive statistics of the participants

A total of 381 clinical nurses opened the survey and consented to participate. The demographic characteristics and the participants’ professional experience of the total sample are shown in Table 1. Of them, 345 completed the survey and were accordingly included in the analysis.

**Table 1.** Demographic characteristics and professional experience of the sample (*N* = 345).

Characteristic	Category	Spain	Chile
		*n* (%)	*n* (%)
Gender, *n* (%)	Female	164 (81.6)	117 (84.2)
Age (SD)	Years, mean	40.3 (11.6)	34.9 (11.3)
Cohabitation^a^	With children	52 (23.2)	35 (23.3)
	Older dependents	29 (12.9)	26 (17.3)
	Chronical illnesses	35 (15.6)	44 (29.3)
	Sick dependents	7 (3.1)	4 (2.7)
	Adolescents	28 (12.5)	19 (12.7)
	Alone	26 (11.7)	18 (7.2)
	Others in addition (parents, adults)	158 (70.5)	91 (60.7)
Professional experience, years	<5	37 (16.5)	53 (48.6)
	5–9	32 (14.2)	12 (11.0)
	10–14	33 (14.7)	8 (7.3)
	15–19	16 (7.1)	12 (11.0)
	>20	106 (47.7)	24 (22.2)

SD: standard deviation.

^a^ Some percentages total more than 100 because nurses selected more than one option.

### Primary aims, the duty to care and well-being

Data related to nurses’ reported well-being, group by country, are presented in Table 2. The mean total scores of the WHO-5 Well-Being Index were 12.5 and 11.8, respectively. Overall, 53.6% of the nurses reported low levels of well-being (WHO-5 score <13).

**Table 2.** Nurses’ well-being (*N* = 345).

Characteristic	Spain (*n* = 206)	Chile (*n* = 139)	Between groups, *p*-value
WHO-5 Well-Being Index, total score,^a^ mean (SD)	12.5 (4.8)	11.8 (4.9)	.246
Low well-being, *n* (%)	103 (50)	82 (59)	.103
High well-being, *n* (%)	103 (50)	57 (41)	

WHO-5: 5-item World Health Organization Well-Being Index; SD: standard deviation.

^a^ On a scale from 0 to 25.

Concerning the aim related to identifying nurses’ agreement with the duty to care even when putting themselves and/or their families at risk, 345 responses were analysed. Among Spanish nurses, 81.1% agreed they have a duty to take care of the sick, whereas 71.9% of Chilean nurses agreed with this statement. Overall, among the 185 nurses who reported low well-being, 51 (27.6%) disagreed that they have a duty to take care of the sick during a pandemic. Concerning the associations between well-being and agreement with the duty to care statement, the chi-square tests revealed that these variables were significantly associated (presented in Table 3).

**Table 3.** Associations between well-being and agreement with the duty to care statement (*N* = 345).

Variables	Spain			Chile			Between groups	
	Disagree	Agree	*p*-value	Disagree	Agree			*p*-value
	Numbers (%)	Numbers (%)		Numbers (%)	Numbers (%)	*p*-value	Total numbers	
Low well-being	20 (19.4)	83 (80.6)	.859	31 (37.8)	51 (62.2)	0.002	185	<0.001^a^
High well-being	19 (18.4)	84 (81.6)		8 (14)	49 (86)		160	
Agreement with statement	39 (18.9)	167 (81.1)		39 (28.1)	100 (71.9)		345	

^a^Chi-square tests.

### Nurses’ confidence, beliefs about managing COVID-19, fears and ethical concerns

Overall, one-fifth of the sample reported a lack of confidence in their abilities to handle the COVID-19 crisis, as shown in Table 4. Regarding concerns and risk perception, a minority of participants reported a lack of concern about contracting COVID-19. In both countries, the majority of nurses reported their perceived risk of infection increased their stress level.

**Table 4.** Concerns and beliefs about the COVID-19 pandemic (*N* = 345).

Variable beliefs about COVID-19	Category	Spain (*n* = 206)	Chile (*n* = 139)
		*n* (%)	*n* (%)
I feel confident in my abilities to handle this COVID-19 crisis	Never	3 (1.5)	3 (2.2)
	Some days	40 (19.4)	32 (23.0)
	More than half	83 (40.3)	54 (38.8)
	Almost every day	80 (38.8)	50 (36.0)
Have you been concerned about contracting COVID-19?^a^	Never	13 (6.3)	8 (5.9)
	Occasionally	88 (42.9)	43 (31.9)
	Very often	51 (24.9)	36 (26.7)
	All the time	53 (25.9)	48 (35.6)
The concern of being infected by coronavirus has increased my stress level	Never	24 (11.7)	15 (10.8)
	Some days	92 (44.7)	53 (38.1)
	More than half the days	32 (15.5)	26 (18.7)
	Almost every day	58 (28.2)	45 (32.4)

COVID-19: coronavirus disease 2019.

^a^ A total of 135 Chilean nurses answered this question.

Table 5 shows the participants’ agreement or disagreement with 15 different ethical concerns about a pandemic. Responses are grouped by country in the form of frequencies and percentages, showing a variation regarding ethical dilemmas in both countries.

**Table 5.** Agreement and disagreement with the ethical concerns about a pandemic (*N* = 345).

Ethical statement	Spain (*n* = 206)		Chile (*n* = 139)		*p*-value
	Agree *n* (%)	Disagree *n* (%)	Agree *n* (%)	Disagree *n* (%)	
Doctors and nurses have a duty to care for the sick even when there are high risks for them or their families	167 (81.1)	39 (18.9)	100 (71.9)	39 (28.1)	.047
HCWs should not receive any special priority during a pandemic, and everyone should have equal access to treatment	56 (27.2)	150 (72.8)	38 (27.3)	101 (72.7)	.97
Every HCW, not just doctors and nurses, has a duty to work during a health emergency even if there are high risks	149 (72.3)	57 (27.7)	74 (53.2)	65 (46.8)	< .001
HCWs must lose their wages if they are unwilling to work during a pandemic	201 (97.6)	5 (2.4)	29 (20.9)	110 (79.1)	< .001
Everyone should pull together during a pandemic	85 (41.3)	121 (58.7)	130 (93.5)	9 (6.5)	< .001
HCWs should be allowed to refuse to work with, or near, infected patients	75 (36.4)	131 (63.6)	89 (64.0)	50 (36.0)	< .001
Professional bodies and unions should offer explicit guidance on whether or not there is a duty to work during a pandemic	92 (44.7)	114 (55.3)	133 (95.7)	6 (4.3)	< .001
People who refuse to work in a time of health crisis should be penalised in some way	91 (44.2)	115 (55.8)	26 (18.7)	113 (81.3)	< .001
People who work during a health crisis should be rewarded in some way	191 (92.7)	15 (7.3)	124 (89.2)	15 (10.8)	.261
My main responsibility is for me and my family. My family will have priority over my work	179 (86.9)	27 (13.1)	115 (82.7)	24 (17.3)	.286
I have to go to work because I could not support myself if I lost any of my wages	204 (99.0)	2 (1.0)	117 (84.2)	22 (15.8)	< .001*
My employer has the responsibility to offer me protective equipment if I have to work during a pandemic	190 (92.2)	16 (7.8)	135 (97.1)	4 (2.9)	.061*
My employer has the responsibility to offer me a vaccination (if available) if I am asked to work during a pandemic	143 (69.4)	63 (30.6)	136 (97.8)	3 (2.2)	< .001*
My employer has the responsibility to offer my family a vaccination (if available) if I am asked to work during a pandemic	157 (76.2)	49 (23.8)	101 (72.7)	38 (27.3)	.456
HCWs must face disciplinary action if they refuse to work during a pandemic	135 (65.5)	71 (34.5)	30 (21.6)	109 (78.4)	< .001

HCW: healthcare worker.

* Fisher’s exact test used when expected *n* is under 5.

## Discussion

Understanding how nurses perceive the ethical challenges around the duty to care for the sick during a pandemic is essential to effectively plan and manage the provision of care. This study identified, for the first time, the ethical challenges relating to the duty to care that nurses in two countries faced due to the COVID-19 pandemic. Furthermore, we described the nurses’ well-being and related associations between well-being and the duty to care.

### The duty to care

During the COVID-19 pandemic, professional nurses have faced conflicting duties. These include the duty to care appropriately for both patients with and without COVID-19 while protecting themselves and their own families from infection. This study focused on coping with these conflicting duties and the associated emotional impact on nurses’ well-being.

Nurses were asked whether doctors and nurses have a duty to care for the sick even when putting themselves and their families at risk. While most nurses agreed they have a duty to take care for the sick, 18.9% of Spanish nurses disagreed with this statement. This percentage was lower than that found in two previous influenza studies (22.1% and 26.5%, respectively)^21,23^ using the same questionnaire. Yet, among Chilean nurses, 28.1% expressed disagreement with the same statement. This percentage is higher than the results from a study conducted in the health services in the United Kingdom where 22.1% of the HCWs did not consider that they had a duty to care as doing so would pose risks to themselves or their families.^21^ Although our findings and available current evidence suggest that more than one-fifth of nurses and HCWs disagreed that they have a duty to take care for the sick during a pandemic,^21^ the numbers seem to be reasonable considering the lack of available PPE when our data were collected. In addition, in line with results from a Palestinian study,^26^ our study found that the majority of the respondents are likely to comply with a commitment to their professional ethics and the duty to care, despite the variation between countries regarding uncertainty, social mobility and poverty. Furthermore, as documented by the qualitative work from the same Palestinian study, the unwillingness to work did not prevent study participants from working. However, if one in five nurses were unwilling to work during a pandemic, it would result in significant challenges to the healthcare system.

### Well-being and the duty to care

Our results showed that Spanish and Chilean nurses self-reported similarly low levels of well-being (50% or more in each country). This finding is in line with several studies that have reported the negative impact of the pandemic on the well-being of nurses and HCWs.^9,29,30^ This low level of well-being can be explained by the increased challenges that nurses faced during the crisis, such as the concern for their safety and increased personal stress, which left them with a conflicting dilemma of whether to care for patients when they feel at risk. This made it difficult for nurses to balance their personal health risk against the risks for the patients and their families if they did not receive proper care from employers. Conflicting priorities might become compounded by fear of outcomes, including those related to the patient’s recovery or demise, how the hospital will cope with the influx of new patients, letting one’s colleagues down, danger to multi-generational and cramped households, and comorbidities that make one more susceptible to severe consequences of infection.

Our findings highlight a key question for nurses: What are a nurse’s duties when their psychological well-being is being negatively impacted? Balancing one’s well-being with the duty to care associated with being a nurse raises important questions, for which the answers have important consequences to both personal health and the healthcare system. In clinical settings, struggling to balance conflicting values over a long period can be challenging^19^ and affect the psychological well-being of nurses.^28^ Navigating this personal challenge in a time of crisis requires support and practical input. In practice, the duty to care imposes nurses to act in the best interests of their patients. However, one has an inherent responsibility to take care of their health,^34^ as well as that of their family. On the contrary, as low well-being was associated with disagreement with the duty to care statement, nurses must be supported to navigate and balance between ethical concerns, duties and well-being. For example, if hospitals could provide alternative accommodation for nurses, childcare facilities, well-spaced on and off duty times, and access to therapists so that doing their duty is not associated with an increased risk to their families, the nurses’ associated ethical dilemma may be resolved. This solution assumes that nurses are in a low-risk group if they were to be infected. Educational and psychological interventions for nurses are recommended as a preparedness measure to cope with the increased stress associated with caring during a pandemic. The nurses’ trust in the protocols for maintaining safety measures on their return home, such as the routines that involve immediate ablutions, changing apparel, distancing and separation from cohabitants, needs to be reinforced with confidence.

### The duty to work or the right to refuse?

This study revealed several ethical challenges related to the right to refuse to care. On the one hand, nurses were asked to agree or disagree with the notion that HCWs should be allowed to refuse to work with, or near, infected patients. Less than half (40%) of the Spanish nurses agreed with this ethical statement, whereas more than 60% of Chilean nurses agreed. Previous studies have reported that majority of nurses (74.7%) do not believe they have the right to refuse to treat patients during the COVID-19 outbreak.^31^ Although the differences in the literature are difficult to explain, when considering that the SARS-CoV-2 virus is contagious, it seems understandable that nurses are concerned about their lives and are afraid of infecting family members. This leads them to agree with the statement. Several studies have supported this explanation by reporting that one of the greatest fears of healthcare personnel during the pandemic is the possibility of infecting others, especially family members.^5,30,35,36^

On the other hand, the responses to the ethical statement regarding the idea that people who refused to work in a time of health crisis should face disciplinary actions or be penalised in some way showed a much larger difference between the two countries. Among Spanish nurses, around half of them agreed to these ethical statements compared with one-fifth of Chilean nurses. This percentage is lower than the results from a study conducted in Spain related to the flu influenza^23^ but similar to the health services in the United Kingdom where less than one-fifth of the HCWs did not consider that they had a duty to care when doing so would pose risks to themselves or their families.^21^

The four bioethical principles of autonomy, justice, beneficence and non-maleficence intend to guide health professionals in making their decisions to protect individual patients’ best interests in clinical medicine. However, these basic biomedical ethical principles do not fully apply amid the COVID-19 pandemic. It may not be possible to ensure both a person-centred focus and a population health focus, since these two concepts might be in opposition. In this context, the principle of non-maleficence should also be applied with a view to deploying nurses in dangerous situations.^37^ The question remains: Should one care for currently infected patients at risk of infecting one’s family? Nurses in both countries reported needing to care for both patients and older cohabiting parents. Furthermore, the need to care for cohabitants with chronic illness was more likely in Chilean nurses than Spanish nurses (29.3% vs 15.6%). In Chile, a lack of social protection for the older people is associated with an increased duty to care for the family. This, together with perceived risk factors such as children at home, can explain why some nurses agreed with a right to refuse. However, having complex challenges regarding the perceived duty to care, as reported by Chilean nurses, contrasts with the Chilean codes of practice, which states that the duty to care is essential. Since healthcare systems around the world are struggling to maintain a sufficient workforce to provide adequate care during the COVID-19 pandemic, if nurses are given the right to refuse to care, this would have several practical and safety implications. Consequently, if nurses are given the right to refuse to care, hospitals will be severely understaffed. On the contrary, if the hospitals cannot provide PPE to protect the nurses, it compromises the safety of the patients and nurses, increasing the moral distress of nurses as well.

### Stress, concerns about safety, responsibility and sanctions

As the COVID-19 pandemic continues, a mounting body of evidence shows that HCWs are facing high levels of stress, concerns for their own safety (or the safety of older people/high-risk cohabitants) and ethical challenges.^5,20,30,31,38^ Therefore, it is not surprising that nurses in both countries agreed equally with the responsibility of the employer to offer protective equipment and vaccinations to the nurses and their families. This finding is consistent with those of previous studies that have reported the overall responsibility of the employer to protect the workers.^21^ Along these lines, if nurses are working in high-risk environments but do not feel sufficiently safeguarded by their employers, they may be less willing to respond to pandemic emergency events at the workplace. This might explain why a large proportion of nurses disagreed when asked if refusing to work during pandemics should be associated with lost pay or some other form of penalty. Of the Spanish nurses, 65.5% agreed that an HCW should be penalised for refusing to work during a pandemic, which is superior to the results reported by Santana 201923 and Damery et al.^21^ that had a lower acceptance of this type of sanction. However, Chilean nurses reported an even lower acceptance of sanctions (20.9%). The implications of accepting penalties or a lack thereof highlight the importance of ethical concerns in the context of pandemics. The accountability and responsibility of not harming must be in balance with the right to work in a safe environment, in which both the health of patients and nurses is protected. As such, nurses’ agreement with the right to refuse to provide care in an unsafe working environment may be reasonable. Considering the survey was completed when there were no vaccines, it seems understandable that nurses did not fully agree with sanctions. However, this finding highlights the presence of considerable concerns and ethical dilemmas that nurses and healthcare systems must be prepared to deal with, namely, the perception of risk, the protection of life and the well-being of nurses, their families and the community they are responsible to care for.

### Limitations and future research

Despite a robust sample size, which attempted to examine diverse nursing populations in two different countries, the data may not be generalisable across time and epidemic context. Moreover, most of the nurses in the sample were females and thus may have had a double burden of care, which may affect the perception of duty to care for the sick. A main, but intended limitation, is that nurses were recruited by snowball sampling. By using only invitations via social media and personal networks, we do not know the response rate of nurses who refused to participate in the study.

Our findings from two different countries suggest that ethical concerns can also conflict with the duty to care for one’s own family. The differences we found between Spain and Chile also illustrate how some of the ethical beliefs differed in a geographical context. Unfortunately, our data are limited to only these two countries. Furthermore, since Chile was previously a Spanish colony, different aspects of these countries’ cultural backgrounds are shared. Thus, it is difficult to speculate about how the national culture could explain some of the differences that were found. Our study did not investigate how different traditional family values, gender role beliefs or cultural values could have influenced the responses to the duty to care. However, our findings encourage future researchers to examine the mechanisms through which traditional gender roles or cultural beliefs could influence nurses’ agreement to the duty to care dilemma. This future research could be clinically important since nurses often work in a multicultural context. Therefore, nurses can experience how cultural or religious beliefs can cause ethical conflicts related to the duty to care for patients. As such, understanding the impact of cultural beliefs, in the context of the duty to care, is required to advance the delivery of nursing in a multicultural pandemic context. Future analysis should explore how agreement to the duty to care statement, in a multicultural context, could be mediated or moderated by the impact of traditional gender role beliefs and well-being.

Finally, the scale used to measure the ethical concerns was initially designed to assess the attitudes and beliefs of healthcare personnel during a hypothetical influenza pandemic. As such, the scale has not been validated in a real-world COVID-19 pandemic context. However, we argue that the scale was most appropriate since it has been used in two previous research studies relating to the duty to care during pandemics.

The insights provided by this study are of high relevance due to the burden of a pandemic and can inform stakeholders and policies across different healthcare settings. Future studies are needed to validate questionnaires measuring the ethical dilemmas during pandemics. When designing future studies, they should control for whether the nurses or their cohabitants are part of high-risk groups.

## Conclusion

The findings highlight increasingly reported ethical dilemmas and concerns related to COVID-19 in two different countries. This shows that the duty to care during a pandemic, results in ethical dilemmas among nurses. When comparing the beliefs associated with the duty to care, most nurses in both countries stated that they would continue working despite the risks to themselves and/or their families. In both countries, nurses were willing to care for the patients, although they reported fears for their own safety, experienced associated stress and low levels of well-being. Among those reporting low well-being, significant differences were found between Spanish and Chilean nurses, as 19.4% and 37.8%, respectively, disagreed that they have a duty to take care of the sick during a pandemic. These results suggest that prompt actions are required to address nurses’ concerns and ethical dilemmas, which are associated with low well-being and their willingness to work. Addressing the ethical concerns of nurses may be valuable when planning care and preparing nurses for working in pandemic settings.
